# Iatrogenic Spinal Subdural Hematoma due to Apixaban: A Case Report and Review of the Literature

**DOI:** 10.1155/2018/4507638

**Published:** 2018-02-19

**Authors:** Alba Colell, Adrià Arboix, Francesco Caiazzo, Elisenda Grivé

**Affiliations:** ^1^Cerebrovascular Division, Department of Neurology, Hospital Universitari del SagratCor, Universitat de Barcelona, Barcelona, Catalonia, Spain; ^2^Department of Neurosurgery, Hospital Universitari del SagratCor, Universitat de Barcelona, Barcelona, Catalonia, Spain; ^3^Department of Neuroradiology, Hospital Universitari del SagratCor, Universitat de Barcelona, Barcelona, Catalonia, Spain

## Abstract

In the last decade, the clinical relevance for developing safer oral anticoagulants prompted the development of new classes of drugs that have shown a lower risk of life-threatening bleeding events as compared to standard warfarin. Nontraumatic spinal subdural hematoma is an uncommon urgent complication that can be associated with the use of these agents. An unusual case of spinal subdural hematoma related to apixaban treatment for nonrheumatic atrial fibrillation is reported here.

## 1. Introduction

Over more than 50 years, warfarin has been the only oral anticoagulant. Warfarin is a vitamin K antagonist with well-documented therapeutic efficacy, despite potential limitations including drug interactions, delayed effect onset of action, narrow therapeutic window, regular measurement of the international normalized ration (INR), and a number of associated hemorrhagic adverse events. For this reason, there has been a great interest in the last decade for developing novel oral anticoagulants to reduce bleeding risks. The new generation of nonvitamin K antagonist oral anticoagulants (NOACs) such as dabigatran, apixaban, and rivaroxaban has more predictable anticoagulant responses and has been shown to be effective in the prevention and treatment of venous thromboembolism and in the prevention of stroke and systemic embolism in patients with nonvalvular atrial fibrillation [1].

Intracranial hemorrhage is one of the most severe complications associated with NOACs as well as posttraumatic spinal hematoma. By contrast, development of a nontraumatic spinal hematoma as a consequence of anticoagulant treatment is an exceptional complication, the natural history of which has been poorly described. Only a few cases of nontraumatic spinal subdural hematoma related to treatment with NOACs have been reported in the literature [2–4]. We report what appears to be the first case of nontraumatic spinal subdural hematoma in a woman with atrial fibrillation during treatment with apixaban. In all previous cases, rivaroxaban was the drug involved in the hemorrhagic adverse effect. The case described here adds further evidence to the development of this life-threatening neurosurgical entity in the context of treatment with the new oral anticoagulants.

## 2. Case Presentation

A previously healthy 75-year-old woman with paraparesis was referred to our hospital for diagnostic studies based on tentative diagnoses of low spinal vascular malformation or secondary spinal bleeding. She had a history of hypertension treated with enalapril 5 mg/daily and atrial fibrillation treated with NOACs. Ten days before admission to the hospital, she presented to the emergency room of a regional hospital with abdominal pain. On physical examination, a spontaneous hematoma 3 × 4 cm in diameter in the left iliac muscle was found. The patient was taking dabigatran at the dose of 150 mg twice a day. Change of dabigatran to apixaban (2.5 mg twice a day) was recommended because of the high cardioembolic risk associated with underlying atrial fibrillation. The dose of apixaban was reduced because of bleeding risk. No other risk factors such as hemostatic or spinal disorders were present. Results of laboratory tests including coagulation tests and renal function tests were unrevealing. Ten days after discharge from the emergency room, she developed multiple cutaneous hematomas in the upper extremities and a sudden onset of predominantly distal bilateral crural motor deficit. She was admitted to the hospital where a predominantly left paraparesis was confirmed. A spinal magnetic resonance imaging (MRI) revealed a hemorrhagic focus suggestive of spinal vascular malformation, which was not confirmed at operation.

**Figure 1:**
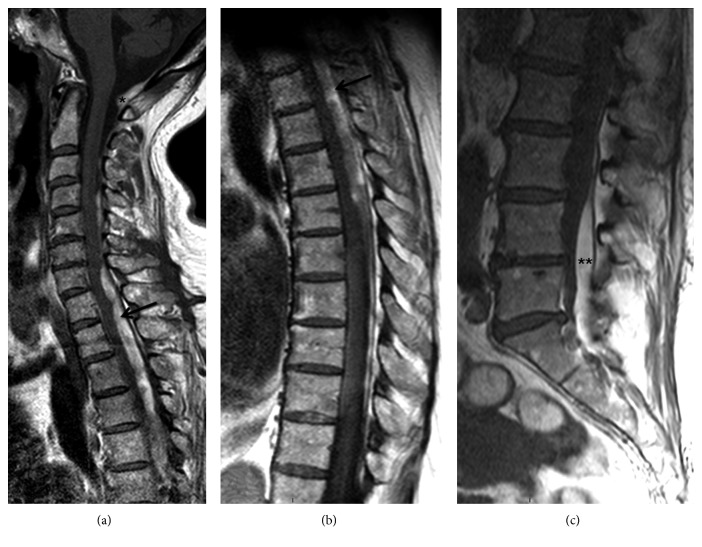
MRI scans: T1 sagittal view of the cervical (a), dorsal (b), and sacral (c) column showing a large subacute intraspinal extramedular hematoma from the craniocervical (^∗^) region to the sacral (^∗∗^) region, with spinal cord compression at the dorsal level (→). It was not possible to study the whole column in one sequence due to clinically silent dorsolumbar scoliosis.

**Figure 2:**
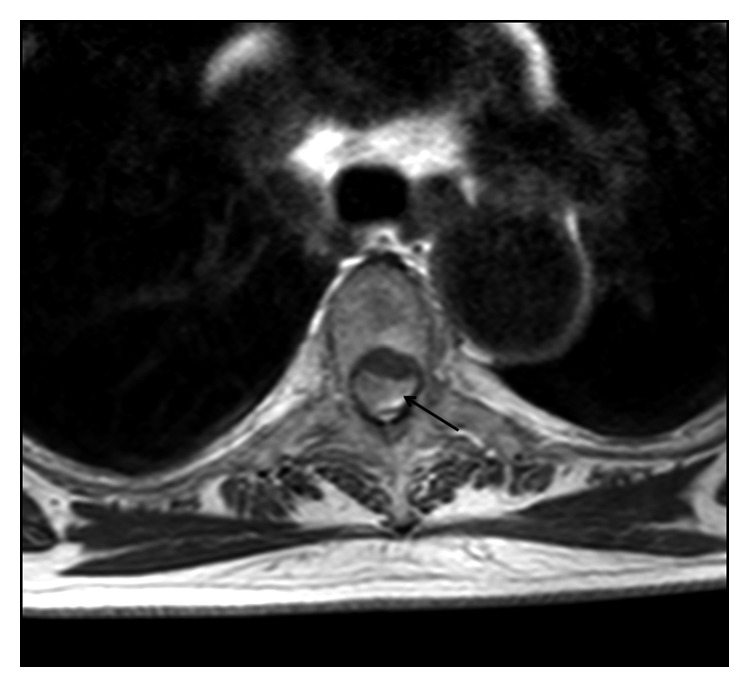
MRI scan: T1 transversal view of the dorsal column. Subacute subdural hematoma (→) with anterior displacement and compression of the spinal cord.

**Figure 3:**
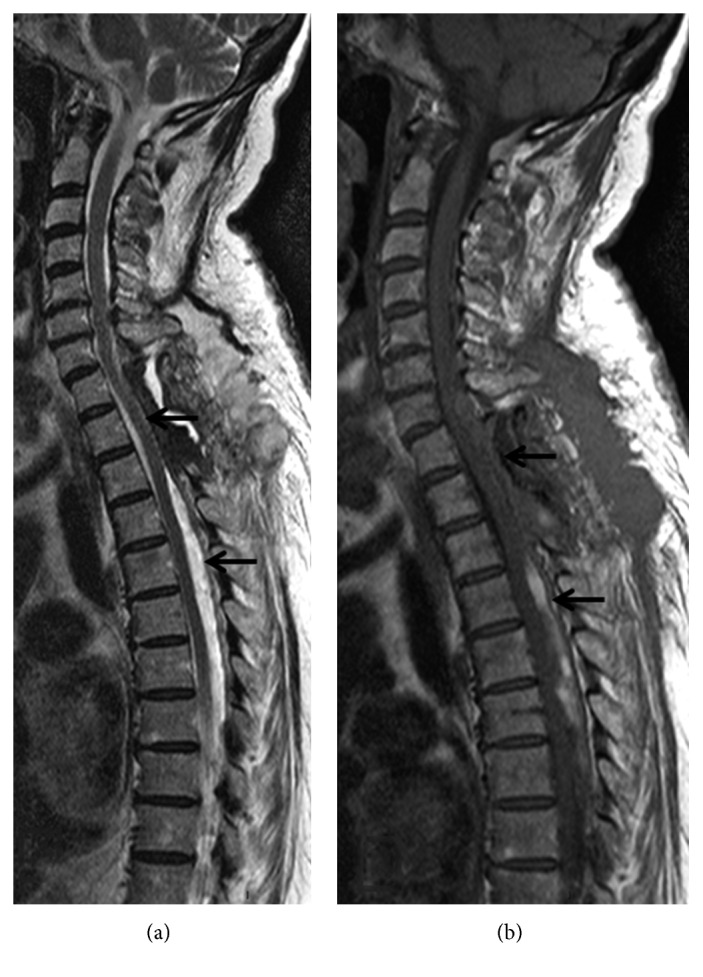
Postsurgical MRI scan. Sagittal views: T2 (a) and T1 (b). Resolution of spinal cord compression in the operated segment (D1–D3) upper arrows, with persistence of cord compression at the D4–D7 level (lower arrows).

On admission to the Department of Neurology of our hospital, neurological examination showed paraparesis, predominantly distal and of the left side, with discrete hyperreflexia, hypoesthesia of the low dorsal deep sensitivity, indifferent left plantar reflex, and normal response on the right side. MRI of the spine revealed a large subdural hemorrhage with discontinuous cervical-dorsal-lumbar-sacral involvement with secondary spinal cord compression but without signs of associated vascular malformation (Figures 1 and 2). The patient was treated surgically, and D1 to D3 vertebral laminectomy was performed. The subdural hematoma was evacuated (36 hours after hospital admission), and the diagnosis of subacute spinal hematoma was confirmed. Postoperatively, she was treated with increasing doses of dexamethasone 12 mg/24 h and standard analgesic medication. The clinical course was satisfactory, but a control MRI scan performed one week after surgery showed persistence of subdural hemorrhage and spinal compression at D4 to D7 segments with resolution of hematoma at D1–D3 (Figure 3). The patient was reoperated and underwent D4 and D7 partial laminectomy and D5 to D7 complete vertebral laminectomy. Postoperatively, paraparesis improved slowly, and the patient was discharged 3 weeks after the last surgical procedure with assisted standing and initiation of walking with aids. Six months after the hospital discharge, the patient's clinical condition had largely improved. She was able to walk alone with the aid of a stick, with mild weakness graded 4/5. A control MRI scan showed D1–D3 and D5–D7 laminectomies without hemorrhagic signs in the spinal cord.

## 3. Discussion

Spinal hematoma is an uncommon clinical entity that can cause sudden and sometimes irreversible neurological impairment if diagnosis and treatment are not promptly established. Apparently, the first clinical case of spinal hematoma was reported by Jackson in 1869 under the term of “case of spinal apoplexy” [5], and the first case of spinal hematoma, which was successfully evacuated by a surgical operation, was reported in 1911 [6]. Depending on its location, the bleeding can be classified as epidural, intradural (subdural), subarachnoid, or intramedullary. Epidural hematomas are the most frequent (75% of cases), with subdural hematomas being extremely rare.

The exact underlying mechanism leading to spontaneous nontraumatic subdural hematoma remains controversial. It is postulated that sudden increases of pressure in the thoracic and/or abdominal cavities can raise the pressure inside the subdural vessels, with rupture of these vessels and subsequent formation of the hematoma [6]. Haines et al. [7] studied the dura-arachnoid junction with electron microscopy. The dura is composed of elongated, flattened fibroblasts and copious amounts of extracellular collagen, whereas the dural border layer (dura-arachnoid junction) is characterized by flattened fibroblasts, no extracellular collagen, extracellular spaces, and few cell junctions. Under normal conditions, there is no evidence of a naturally occurring space at the dura-arachnoid junction, but a space may appear at this point subsequent to pathological/traumatic processes that result in tissue damage with a cleavage opening of the structurally weakest plane in the dural border cell layer. Therefore, subdural hematomas should be considered intradural or hematomas of the dural inner cell border.

Spinal subdural hematomas, as in the case reported here, are usually secondary to vascular lesions, underlying tumors, or result from invasive procedures, such as lumbar puncture and spinal anaesthesia (or even acupuncture) [8], associated with blood dyscrasias and coagulation defects [9]. In a review of 106 cases of nontraumatic acute subdural spinal hematoma, there was a defect in the hemostatic mechanism in 57 patients (54%) and a history of spinal puncture in 50 patients (47%) [10]. Primary or spontaneous nontraumatic spinal hematomas are very uncommon.

A few cases of spinal hematoma directly related to anticoagulant treatment have been reported, most of them presenting as epidural hematomas [11]. Only three cases of spontaneous spinal subdural hematoma associated with the use of rivaroxaban have been reported in the literature [2–4], but to our knowledge, no case due to apixaban has been described. Salient features of these cases including the present case are summarized in Table 1.

Acute and sudden onset of symptoms, with dorsolumbar pain followed by symptoms of spinal cord compression, is characteristic clinical manifestations [11], although multiple neurological deficits have been reported, such as sensory and motor deficits, sphincter dysfunction, and Brown-Séquard syndrome. Our patient did not have dorsal pain, and her clinical picture was mostly a mixed motor-sensory deficit.

MRI is the technique of choice for diagnosis and preferable to computed tomography (CT) [12]. MRI has a high sensitivity for defining the type of bleeding and assessing the craniocaudal extension of the hematoma. Changes in the intensity of MRI signal after development of spinal hematoma are similar to those of cerebral hematomas, although it seems that metabolism of hemoglobin in the spinal canal is faster than that in the intracranial space. Thus, T1-isointense foci and T2-hyperintense lesions due to the presence of oxyhemoglobin can be seen during the first 24 hours, followed by hypointense T1 and T2 signals on the second and third days and subsequent greater signal intensity in both sequences due to the appearance of methemoglobin. Cervicodorsal and dorsolumbar levels are the most frequently affected regions, with predominantly cervical and dorsal involvement in young patients, whereas dorsolumbar involvement is more common in adults. Our patient showed extensive involvement especially in the D1 to D7 region.

The management of nontraumatic spontaneous spinal subdural hematoma has not been definitively established, probably because of the rarity of this condition. The management usually includes emergent surgical evacuation and discontinuation of NOACs. Conservative management may be considered in cases with mild neurological deficits, early spontaneous recovery, or high surgical risk [4]. Prognosis is poor in the presence of associated subarachnoid hemorrhage with spinal arachnoiditis as a potential complication [13].

## 4. Conclusion

Nontraumatic spontaneous spinal subdural hematoma is a potential complication of the use of apixaban, and this appears to be the first case reported in the literature. This entity should be distinguished from spinal subarachnoid hemorrhage, spinal epidural hemorrhage, or intraspinal hemorrhage [14]. Physicians should be aware of the increasing incidence of severe bleeding complications associated with the increasing use of NOACs. Serious spinal hematomas secondary to rivaroxaban and apixaban therapy are a devastating complication for the patient and the family. Recovery of the neurological deficit depends on prompt diagnosis with MRI scans and early surgical evacuation of the hematoma.

## Figures and Tables

**Table 1:** Characteristics of the cases of nontraumatic spontaneous spinal subdural hematoma secondary to NOACs.

Author [reference]	Sex, age	Topography	Type of drug, dose	Indication of anticoagulation	Symptom	Treatment	Outcome
Castillo et al. [2]	M, 69 yrs	Thoracolumbar	Rivaroxaban, 20 mg/day	Atrial fibrillation	Lumbar pain, paraplegia, sphincter dysfunction	Cervical and lumbar drainage	No improvement
Dargazanli et al. [3]	M, 72 yrs	Thoracic	Rivaroxaban, 20 mg/day	Atrial fibrillation	Acute interscapular pain, paraplegia	Prothrombin complex, surgery	No improvement at 6 months
Zaarour et al. [4]	F, 58 yrs	Cervicothoracic	Rivaroxaban, 20 mg/day	Atrial fibrillation	Acute interscapular pain, weakness of lower extremities	High-dose steroids, surgery	Important improvement but not complete
Present case	F, 75 yrs	Cervical-dorsal-lumbar-sacral	Apixaban 2.5 mg/12 h	Atrial fibrillation	Acute paraparesis (left side predominant)	High-dose steroids, two surgical operations	Partial improvement at 1 month after the second operation
